# A Non-Resonant Kinetic Energy Harvester for Bioimplantable Applications

**DOI:** 10.3390/mi9050217

**Published:** 2018-05-05

**Authors:** Mustafa İ. Beyaz, Hacene C. Baelhadj, Sahar Habibiabad, Shyam S. Adhikari, Hossein Davoodi, Vlad Badilita

**Affiliations:** 1 Department of Electrical and Electronics Engineering, Antalya Bilim University, Antalya 07190, Turkey; hacene.baelhadj@std.antalya.edu.tr; 2 Graduate School of Natural and Applied Sciences, Middle East Technical University, Ankara 06800, Turkey; saharhabb@gmail.com; 3 Spin & Photon Applications (SPA) Lab, Institute of Microstructure Technology, Karlsruhe Institute of Technology (KIT), Karlsruhe 76344, Germany; shyam.adhikari@kit.edu (S.S.A.); hossein.davoodi@kit.edu (H.D.); vlad.badilita@kit.edu (V.B.)

**Keywords:** energy harvesting, implantable devices, magnetic harvesters, MEMS

## Abstract

A linear non-resonant kinetic energy harvester for implantable devices is presented. The design contains a metal platform with permanent magnets, two stators with three-dimensional helical coils for increased power generation, ball bearings, and a polydimethylsiloxane (PDMS) package for biocompatibility. Mechanical excitation of this device within the body due to daily activities leads to a relative motion between the platform and stators, resulting in electromagnetic induction. Initial prototypes without packaging have been fabricated and characterized on a linear shaker. Dynamic tests showed that the friction force acting on the platform is on the order of 0.6 mN. The resistance and the inductance of the coils were measured to be 2.2 Ω and 0.4 µH, respectively. A peak open circuit voltage of 1.05 mV was generated per stator at a platform speed of 5.8 cm/s. Further development of this device offers potential for recharging the batteries of implantable biomedical devices within the body.

## 1. Introduction

Bioimplantable devices are attracting much attention, as they provide effective solutions for monitoring and treatment of diverse health problems. Examples for already-developed devices include pressure sensors, pacemakers, muscle stimulators, and glucose sensors that can control and monitor diseases ranging from heart-related disorders to diabetes. The spectrum of such devices is expected to continuously expand by targeting new health problems in the near future. Depending on the specific application, bioimplantable devices require electrical power on the order of 10 µW–10 mW [1]. Currently, the power demand is met by electrochemical batteries [2]. This mandates repeated surgeries to replace depleted batteries, and leads to patient discomfort as well as inflammation risks associated with the surgical procedures. Energy harvesters, either as continuous battery rechargers or as total battery replacements, can be a promising solution to overcome these problems and enable a life-time operation for implantable devices. Accordingly, significant efforts have been dedicated to the development of energy harvesting technologies.

Numerous energy harvesting devices with different power generation/transfer mechanisms have been demonstrated. Among these, wireless power transfer is probably the most established, and is becoming an integral component in recently developed biomedical devices. This technique is essentially based on electromagnetic coupling of an ex vivo transmitter coil and an in vivo receiver coil, so that power is transferred inside the body when the external transmitter is energized [3,4]. Several devices using this technique have been presented for various applications including deep brain stimulation [5] and blood flow sensing [6]. The major drawbacks of wireless power transfer have been reported to be possible tissue heat-up [4] and patient discomfort due to the presence of the external transmitting device, which has to be within the close proximity of the implant [1]. The dependence to the external transmitting device also prohibits this technique from being a totally untethered solution for powering implantable devices. Alternatively, thermoelectric generators based on the Seebeck effect have been utilized to convert temperature gradient around the skin into electrical power. Temperature differences at skin proximity under realistic and comfortable conditions are around 10 K. Hence, open circuit voltage and output power for thermal generators are typically on the order of millivolts and nanowatts, respectively [7,8,9]. Serial combination of many thermocouples have been shown to increase the voltage and power by several orders of magnitude [10,11,12,13,14]. The power output of thermal generators heavily depends on external temperature. In addition, the thermal gradient demand of these generators generally confines their application in the skin’s vicinity. This severely limits their use for deep implants, or mandates additional and undesirable cabling for power delivery.

Considering the mechanical efficiency of human beings is around 15–30% [15], it is quite feasible and expedient to convert the wasted mechanical power in human motion into electrical power. Accordingly, a large number of studies have been focused on generating power from walking, running, and the motion of body parts using piezoelectric, electrostatic, and electromagnetic transductions. Piezoelectric transduction mechanism has been used for in vivo power generation by embedding piezoelectric sheets (PZT) around muscles and joints to convert the strain into electricity. A piezoelectric generator was embedded around a rabbit’s quadriceps, and produced around 2 μW output power under 40 N force [16]. Higher levels of power generation were also reported in similar studies, where the generator size exceeded 1 cm^3^ and was placed outside the body [17,18,19,20]. Similar to thermal generators, piezoelectric generators are location-dependent and can only be implemented around the moving body parts or joints. In comparison, electrostatic and electromagnetic devices are less dependent on the implantation site and can harvest energy from various types of human motion by utilizing their inertia. This also allows them to be incorporated into the implanted biomedical device. The power generated by electrostatic harvesters is generally one order of magnitude less than electromagnetic counterparts [1], and typically below 100 μW [21,22,23,24]. On the other hand, electromagnetic devices developed to harvest heart beats [25,26], abdominal wall movements [27], knee motion [28,29], and walking [30,31] were reported to generate power up to milliwatts depending on their size and available mechanical energy.

Although developed to harvest a variety of body motions, the electromagnetic devices demonstrated so far have generally been tailored for a specific implantation site on the body. This significantly decreases the output power if the biomedical device together with the harvester is implanted on a different location for medical reasons, or if the harvester is to be coupled with a new type of biomedical device operating on another part of the body. In this work, we present a linear electromagnetic device that can harvest the kinetic energy of the body regardless of where it is situated. The main device feature facilitating location independence is that the design employs an untethered platform free to move on ball bearings. Hence, a slight movement of the implantation site triggers platform displacement and results in voltage induction and power generation. The non-resonant device design allows for capturing any type of body motion such as walking, running, twisting, etc., regardless of the frequency. This also eliminates the need for large proof masses to collect low-frequency excitations, and allows for the device volume to be less than 1 cm^3^ and implantable. The location independence, non-resonant nature, and small footprint features enable this harvester to be integrated into a variety of bioimplantable devices targeted for different parts of the body. In this paper, we report the design, simulations, fabrication and test results for the first prototypes, as well as some aspects for further improvement.

## 2. Design and Simulation

**Figure 1:**
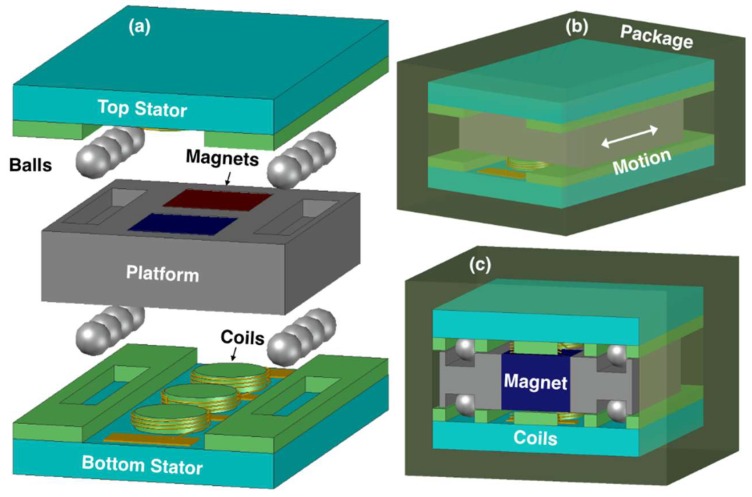
(**a**) Exploded view of the device; (**b**) packaging scheme; (**c**) cut-away view.

A general schematic of the device is shown in Figure 1a. The design is comprised of two stators with helical coils, and a metal platform with two embedded permanent magnets. The platform is free to move along one axis on Si_3_N_4_ balls (Ø = 1 mm), which are located on four sides of the platform. The balls are sandwiched between the trenches on the platform and stators, and are used to obtain low friction during platform displacement. The stators are attached inside an encapsulating polydimethylsiloxane (PDMS) package, providing biocompatibility (Figure 1b,c), such that they are effectively anchored to the body through the package. The device is envisioned to be implanted in the body parallel to the ground. In this configuration, slight changes in the body posture due to any type of body movement result in instantaneous accelerations along the device axis as well as horizontal imbalances, leading the platform to move over the balls rolling in the trenches. This creates a relative motion between the magnets and the coils, which is converted into voltage and power generation through electromagnetic induction.

**Figure 2:**
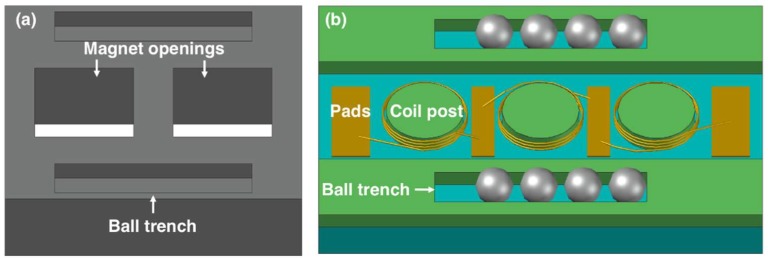
Close-up view of the (**a**) platform, and (**b**) stator.

The close-up view of the platform is shown in Figure 2a. It consists of an aluminum frame with two square-shaped openings for magnet integration. The platform is machined to have four trenches to accommodate the balls. The magnets were selected to be NdFeB for their high remanent flux density of 1.4 T, and were inserted into the openings in alternating polarity.

Each stator contains three separate coils on a pyrex substrate (Figure 2b). The coils are made of 50-µm-thick copper wires wound around cylindrical posts in multiple turns and in helical configuration. Three coils on each stator are connected in series through electrical pads for additive voltage induction. In addition, winding directions of the coils and the relative positions of the stators are designed such that both stators can provide in-phase voltages for further serial connection and voltage amplification. Similar-sized trenches are also defined on the stators. The device is assembled with four balls on each trench, allowing a 2.5-mm-long travel distance for the platform and a 50-µm air gap between the coils and the magnets. The dimensions of the platform and the stators are selected to yield an overall volume of less than 1 cm^3^, and are provided in Table 1.

**Figure 3:**
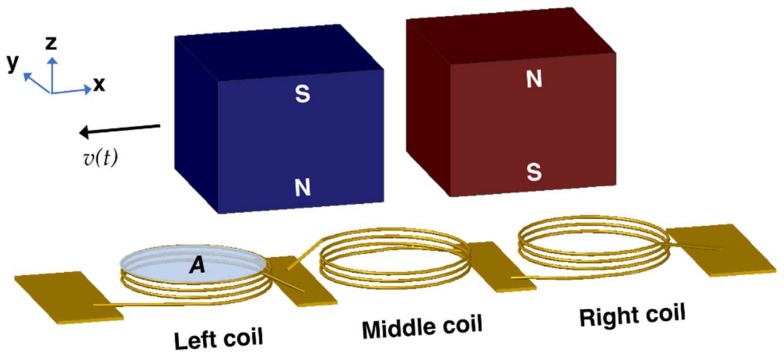
Schematic showing the relative displacement between magnets and coils.

The relative displacement between magnets and coils during platform actuation is illustrated in Figure 3. The vertical component of the magnetic flux density, *B_Z_*, acting on the coils with area *A* changes with time and results in voltage induction dictated by:

(1) $$V_{OC}\left(t\right)=N\times A\times \frac{dB_{Z}}{dt}$$

where *V_OC_*(*t*) and *N* are the open circuit voltage amplitude and number of turns per coil, respectively. When all three coils on a stator are serially connected, the total voltage *V_T_*(*t*) becomes:

(2) $$V_{T}\left(t\right)=V_{OC,L}\left(t\right)-V_{OC,M}\left(t\right)+V_{OC,R}\left(t\right)=N\times A\times \left(\frac{dB_{Z,L}}{dt}-\frac{dB_{Z,M}}{dt}+\frac{dB_{Z,R}}{dt}\right)=N\times A\times \frac{dB_{Z,T}}{dt}$$

where the subscripts represent the left (*L*), middle (*M*), and right coils (*R*) and the total (*T*), respectively. The minus sign in the equation is due to the opposite winding direction of the middle coil for additive voltage induction. The terms in (2) should be determined to estimate the open circuit voltage amplitude per stator. The number of turns in this initial study was selected to be *N* = 12 to decrease fabrication complexities, while the diameters of the coils were designed to be 2 mm, leading to *A* = π mm^2^. The final term depends on the variation of the total flux density on the coils as the platform moves along *x* axis (Figure 3), as well as on the velocity of the platform *v*(*t*). In this respect, the d*B_Z,T_*/d*t* can be written as:

(3) $$\frac{dB_{Z,T}}{dt}=\frac{dB_{Z,T}}{dx}\times \frac{dx}{dt}=\frac{dB_{Z,T}}{dx}\times v\left(t\right)$$

**Figure 4:**
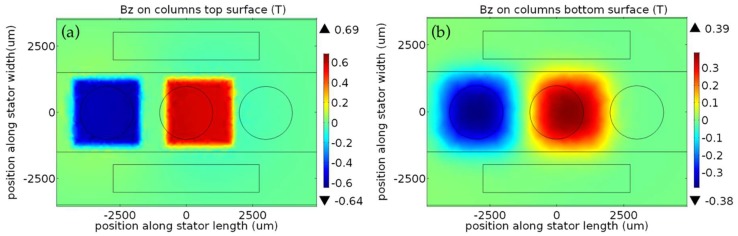
The magnetic flux density on the (**a**) top and (**b**) bottom of the coil post when the platform is in the leftmost position.

**Figure 5:**
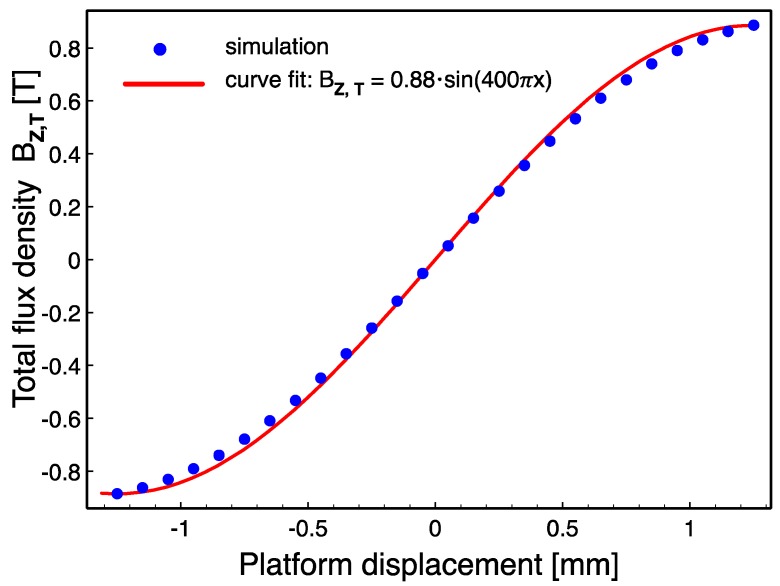
The variation of total flux density, *B_Z_*_,_*_T_* with respect to platform displacement.

The magnetic flux density of a rectangular magnet at a distance *z* from the magnet surface can be written as:

(4) $$B\left(z\right)=\frac{B_{r}}{\pi }\left[\arctan\left(\frac{LW}{2z\sqrt{4z^{2}+L^{2}+W^{2}}}\right)-\arctan\left(\frac{LW}{2\left(D+z\right)\sqrt{4{\left(D+z\right)}^{2}+L^{2}+W^{2}}}\right)\right]$$

where, *B_r_* is the remanence flux density, and *L*, *W*, *D* are the length, width, and thickness of the magnet, respectively [32]. To find the *B_Z,T_* as a function of *x* in Equation (3), this expression should be evaluated at each point in the coil volume and averaged for different platform locations along the travel distance. Instead, the AC/DC module of the COMSOL (COMSOL Inc., Stockholm, Sweden) finite element software was used, which applies Gauss law to the given geometry and calculates the flux density within the coils. Figure 4 shows an example simulation result, and demonstrates the distribution of the vertical component of the flux density on the top (Figure 4a) and bottom (Figure 4b) of the coil posts when the platform is at the leftmost end of the 2.5-mm-long travel distance (*x* = −1.25 mm). The slight misalignment between the magnets and the coils is due to the platform manufacturing tolerances taken into account during simulations. As can be clearly seen from the results, there is a significant difference between the flux densities at the top and bottom of the coils. Therefore, the effective vertical flux density *B_Z_* on any coil is found by taking the volume average as:

(5) $$B_{Z}=\frac{1}{V}\int B_{Z}\left(x,y,z\right)dV$$

where *V* is the corresponding coil volume. The simulations are repeated in 0.1 mm displacement steps and *B_Z,T_* is then found by using (2) at each step. The variation of *B_Z_*_,*T*_ with respect to the platform displacement and a sinusoidal curve fit are shown in Figure 5. Finally, assuming the platform moves at a speed of *v* m/s, the platform displacement can be written as *x*(*t*) = *v* × *t*, where *x =* 0 corresponds to *t* = 0. Combining all of the above together with (2), the open circuit voltage amplitude per stator becomes:

(6) $$V_{T}=12\times \pi \times 10^{-6}\times 400\pi \times 0.88538\times \cos\left(400\pi vt\right)\times v=0.042\times v\times \cos\left(400\pi vt\right)$$

yielding a sinusoidal voltage with an amplitude of 42 mV and a frequency of 200 Hz at 1 m/s platform speed.

Previous demonstrations of similar-sized helical coils show that their reactance is negligible compared to coil resistance below MHz-range frequencies [33,34], which are far beyond the expected frequency of the induced voltage. Accordingly, the coils can be modelled as pure resistors with resistance *R* as:

(7) $$R\approx N\frac{\rho \left(2\pi r\right)}{A_{w}}$$

where *ρ* is the resistivity of the copper, *r* is the 1 mm post radius, and *A_w_* is the wire cross sectional area. For the 12-turn coils wound with 50-µm-thick wires, the total resistance of three coils in series on a stator is calculated to be 1.95 Ω. Finally, the maximum rms output power per stator on a matched load can be found by:

(8) $$P_{rms}=\frac{{\left(V_{T,peak}\right)}^{2}}{8R_{S}}$$

where, *V_T_*_,_*_peak_* is the peak open circuit voltage amplitude and *R_S_* is the total coil resistance per stator, leading to 113 µW output power at 1 m/s platform speed. When two stators are connected in series, the open circuit voltage and power become 84 mV and 226 µW at this speed, respectively.

## 3. Device Fabrication

**Figure 6:**
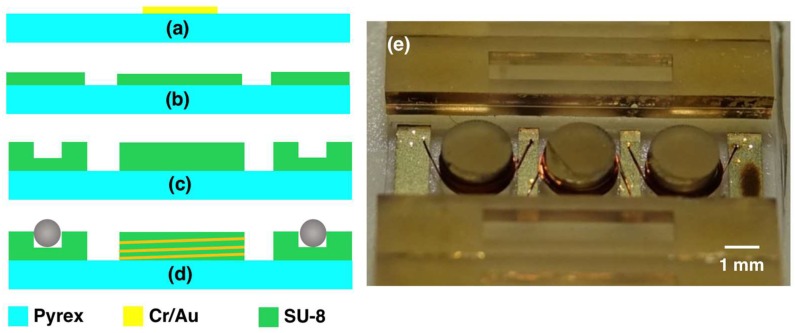
(**a**–**d**) Microfabrication steps; (a) and (b–d) are drawn using different cross-sections of the stator. (**e**) a picture of the fabricated stator.

Stators are fabricated on 500 µm-thick pyrex substrates. Initially, 10 µm-thick electrical pads are defined on the wafer using electroplating process. This thickness is necessary for the adhesion of the wires to the pads during the subsequent wirebonding step. Accordingly, 10 nm Cr and 50 nm Au layers are thermally evaporated on the wafer to serve as a seed layer. Next, SU-8 3025 resist is patterned on the wafer as a mold, and the Au layer is electroplated. The SU-8 mold is then stripped in oxygen plasma environment and the seed layer is etched using gold and chromium wet etchants, respectively (Figure 6a). To decouple the coil height from the trench depth and achieve longer coils, the coil post and ball trench are fabricated using two SU-8 lithography steps. In the first step, 4 mL of SU-8 2150 is statically dispensed onto the wafer at 80 °C and the solvent is removed by performing the softbake at 95 °C for 4 h, resulting in 350 µm layer thickness. The photoresist is then patterned with a dose of 800 mJ/cm^2 ^using an i-line filter. This first layer defines the coil posts and elevates the bottom of the trench on the two sides of the wafer (Figure 6b). In the second step, another 5 mL of SU-8 2150 is dispensed at 80 °C immediately after the exposure of the first layer, and softbaked at 95 °C for 5 h to yield 450 µm layer thickness. This softbake step also acts as a post exposure bake (PEB) for the first layer. The resist is exposed to a dose of 1800 mJ/cm^2^, and a final PEB is applied. The substrate is slowly cooled to room temperature over a period of 8 h to control the thermal stress on the substrate. This second layer further increases the coil post height while defining the ball trench (Figure 6c). Accordingly, the coil post height and trench depth become 800 µm and 450 µm, respectively. Next, the wafers are diced into individual stators using a dicing saw, and copper wires are wound around the coil posts 12 times using a wire bonder (Figure 6d). The picture of a fabricated stator is shown in Figure 6e. After stator fabrication, two wires are soldered to the stator terminals for electrical connection and balls are placed in the trenches using a pair of tweezers.

The platform is computer numerical control (CNC)-machined out of aluminum with a tolerance of 100 µm in all directions. Finally, NdFeB magnets are manually inserted into the openings on the platform. The manual assembly of the balls and the magnets, as well as the device assembly described below can be performed using a pick-and-place-type robotized platform in future implementations. In addition, PDMS packaging was not implemented in these initial prototypes, and left for second-generation devices.

## 4. Testing and Discussions

**Figure 7:**
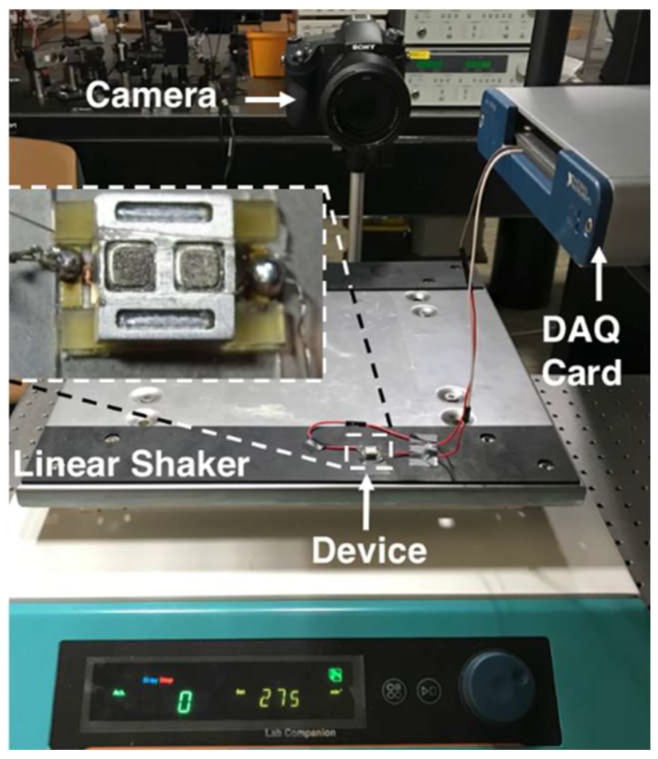
Picture of the test setup and the device under testing.

A test setup was built to characterize the fabricated harvester prototypes. This setup employs a linear shaker to actuate the device, a DSC-RX10M3 (Sony Inc., Tokyo, Japan) high-speed camera and Kinovea (kinovea.org) motion tracking software, and a data acquisition card together with the Labview (NI Corporation, Austin, TX, USA) software to capture the induced voltage between stator terminals. The device was assembled using a single stator fixed to the shaker, while the platform on top was free to move on the balls. A picture of the test setup is shown in Figure 7.

Initially, the resistance and inductance of the three coils in series were measured using an E4980A LCR meter (Keysight Inc., Santa Rosa, CA, USA). The total resistance was measured to be 2.2 Ω, which is very close to 1.95 Ω predicted by (7). The inductance was measured to be 0.4 µH, yielding the same magnitude of reactance at 0.9 MHz. Therefore, the coils can be accepted to be purely resistive, as initially assumed.

**Figure 8:**
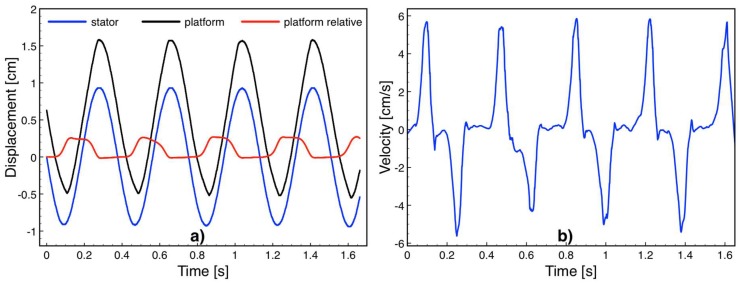
(**a**) Stator and platform displacement during actuation at 2.67 Hz; (**b**) velocity of the platform with respect to the stator.

The friction force acting on the platform is an important factor impeding voltage induction. To determine the friction force, the stator was actuated using the shaker and the platform motion was recorded using the camera at 1000 fps. The actuation conditions were set within the limits of the linear shaker. Figure 8a shows the stator and platform displacements as well as the relative displacement between the two obtained by Kinovea software while the device was driven into harmonic motion at 160 rpm (2.67 Hz) over 2 cm travel distance. The relative platform speed shown in Figure 8b was obtained by numerically differentiating the position versus time data, exhibiting a maximum value of 5.8 cm/s. Since the actuation distance was longer than the 2.5 mm-long platform travel distance, the platform instantly switches between the leftmost and rightmost points on the device as the stator approaches to the two ends of 2-cm-long actuation. Accordingly, the relative motion was rather impulsive and reached the highest velocity when the platform and stator are moving in opposite directions. The deceleration at this point was solely due to the friction force *F_f_*, and was calculated by *F_f_* = *m* × *a*, where *m* is the platform mass and *a* is the deceleration obtained by numerically differentiating the velocity versus time data. The platform mass was measured to be 0.35 g and the deceleration values were on the order of 1.7 m/s^2^, leading to a 0.6 mN friction force on the bearings.

**Figure 9:**
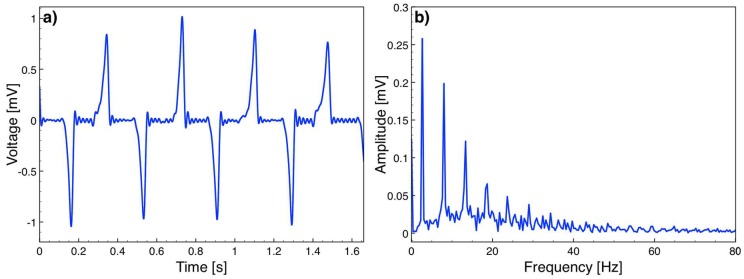
(**a**) Open circuit voltage vs time at 2.67 Hz actuation; (**b**) frequency spectrum of the voltage.

While the actuation was ongoing, the open circuit voltage induced between the stator terminals was sampled at 400 kHz and recorded using the data acquisition card and Labview. The data was processed in Matlab (Mathworks Inc., Natick, MA, USA) using a low pass filter to remove the noise at 50 Hz and higher frequencies. Figure 9 plots a sample open circuit voltage versus time data and the frequency spectrum, showing peaks at 2.67 Hz and its harmonics. Note that Figure 8b and Figure 9a do not represent the same time window as the camera and Labview could not be synchronized in the current test setup. However, as the velocity peaks are in the middle of the triangular velocity profile, the platform reaches the maximum velocity around *x* = 0 (Figure 5), which corresponds to *t* = 0 in (6). Hence, the velocity peaks and voltage peaks occur simultaneously. The repeated experiments showed that the maximum voltage amplitude was 1.05 mV corresponding to the highest platform speed of 5.8 cm/s. The voltage at this speed was expected to be 2.43 mV according to (6). Smaller values are believed to be mainly due to imperfect magnetization of the magnets, specifically around the magnet edges. In addition, the air gap in the simulations was taken as the distance between the magnets and coil posts, however the actual air gap is slightly larger, since the coils winding cannot reach up to the top of the post. Both effects reduce the magnetic flux density, and hence the induced voltage on the coils. The voltage waveform also deviated from a pure cosine form mainly as the velocity profile of the platform was not constant over time.

The first prototypes reported here were not tested for power, due to the level of voltages obtained in the experiments, where excitation on a human body is imitated with a linear shaker. However, daily activities of humans are generally around similar frequencies but with a larger amplitude than the 2 cm used in the experiments. In addition, activities such as walking and running can further trigger abrupt displacements on the platform when the device is implanted. Accordingly, the platform speed is expected to reach walking speeds on the order of 1 m/s, leading to an approximately 20-fold increase in the open circuit voltage. At this speed, collisions between the balls and trench walls at the end of 2.5 mm platform travel can reverse the platform motion and result in continued excitation. In parallel, several improvements can be made in the device development to further increase the voltage and output power. The voltage is directly proportional to the number of coil turns dictated by (2). In this respect, the coil winding process can be enhanced to allow for more turns around a coil post. Alternatively, the same coil post can be wound with different set of coils having separate pads, which can be connected in series. Both strategies would yield a higher number of turns per coil post and improve voltage induction. Equations (2) and (3) also show that the voltage is proportional to the average magnetic flux density within the coils as well. The flux density can be boosted by magnetizing the permanent magnets using an in-house built setup that can provide a uniform high magnetic field over a large surface. This can ensure that the magnet edges are magnetized up to the remanence and magnets are fully utilized. Finally, the use of two serially connected stators embedded in the designed PDMS package together with all the improvements described above is expected to bring the voltage and power levels on the order of 0.1 V and 0.1 mW on a matched resistive load, respectively.

## 5. Conclusions

We presented an implantable linear harvester that can convert the available kinetic energy in body movements into electrical energy through electromagnetic induction. When implanted horizontally in the body, the untethered actuation scheme allows for exploiting all types of body motion, regardless of the frequency and implantation site. The first prototypes were successfully fabricated with a limited number of turns and tested on a linear shaker. Mechanical tests revealed a 0.6 mN friction force in the bearings. The inductance of the three coils on a stator was shown to be 0.4 µH and negligible compared to the 2.2 Ω coil resistance. Open circuit voltage tests demonstrated that 1.05 mV was induced between stator terminals at a platform speed of 5.8 cm/s. Several potential improvements in the device design and development are discussed. With these improvements, the device is expected to generate an output power on the order of 0.1 mW when triggered by normal body activities. The energy harvester presented in this work enables continuous battery recharging within the body for a variety of implantable biomedical devices, and offers potential for eliminating surgical procedures for battery replacements.

## Figures and Tables

**Table 1:** Dimensions of the device components and features.

Platform		Stator	
Length	7.5 mm	Length	11.5 mm
Width	7 mm	Width	7.5 mm
Thickness	2 mm	Thickness	1 mm
Trench length	5 mm	Trench length	5.5 mm
Trench width	1.05 mm	Trench width	1.05 mm
Trench depth	0.5 mm	Trench depth	0.45 mm
Travel distance	2.5 mm	Number of balls	4
Magnet length	2.5 mm	Coil post height	0.8 mm
Magnet width	2.5 mm	Coil post diameter	2 mm
Magnet thickness	2 mm	Number of coil turns	12
Total volume with packaging	0.98 cm^3^	Wire diameter	50 µm
